# Combinations of Genetic Variants Occurring Exclusively in Patients

**DOI:** 10.1016/j.csbj.2017.03.001

**Published:** 2017-03-10

**Authors:** Erling Mellerup, Gert Lykke Møller

**Affiliations:** a Laboratory of Neuropsychiatry, Department of Neuroscience and Pharmacology, Faculty of Health, University of Copenhagen, Denmark; b Genokey ApS, ScionDTU, Technical University of Denmark, Hoersholm, Denmark

**Keywords:** Genetic variants, Polygenic disorder, Combinations of genetic variants, Patient-specific combinations

## Abstract

In studies of polygenic disorders, scanning the genetic variants can be used to identify variant combinations. Combinations that are exclusively found in patients can be separated from those combinations occurring in control persons. Statistical analyses can be performed to determine whether the combinations that occur exclusively among patients are significantly associated with the investigated disorder. This research strategy has been applied in materials from various polygenic disorders, identifying clusters of patient-specific genetic variant combinations that are significant associated with the investigated disorders. Combinations from these clusters are found in the genomes of up to 55% of investigated patients, and are not present in the genomes of any control persons.

## 1 Introduction

A polygenic disorder is caused by the combined effects of multiple genes. Within this concept, it is implicit that the combination of genetic variants constituting or contributing to the basis of the disorder will not normally be present in healthy subjects who are not genetically related to the patients. Although many common disorders are considered to be polygenic, no genetic variant combination has been identified as clearly being basis of a polygenic disorder. This is largely because very few genetic variants were known until recently. Researchers have now identified a huge number of genetic variants, facilitating the search for combinations. However, the large number of known variants gives rise to an immense number of combinations, presenting mathematical, statistical, and computational challenges.

The theoretical number of possible combinations can be calculated using the formula *n*! / *r*!(*n* − *r*)!, where *n* represents the number of genetic variants analyzed in a study, and *r* represents the number of genetic variants per combination. If the genetic variants are SNP genotypes, the formula is n! / r!(n − r)! × 3^r^. Thus, if 100 variants are analyzed, the theoretical number of 10-variant combinations would be 1.73 × 10^13^. Likewise, if 500,000 SNPs are analyzed, there would theoretically be 2.3 × 10^12^ two-variant combinations and 3.4 × 10^18^ three-variant combinations. However, it is not yet known how many genetic variants are present in the combinations related to any polygenic disorder; there must be at least two, but the upper limit is uncertain.

Notably, analyses of variant combinations are also affected by the unclear genetic homogeneity or heterogeneity of polygenic disorders. While a polygenic disorder showing genetic homogeneity would be associated with only one combination of genetic variants, a genetically heterogeneous polygenic disorder would be associated with multiple different genetic variant combinations. In the latter circumstance, the number of responsible genetic variant combinations could be small and correspond to a limited number of genotypes, or could potentially be very large. Since the total number of combinations could be thousands of billions, if a disorder is associated with even just a small percentage of these combinations, this could correspond to billions of combinations. Thus, it cannot be excluded that, for some polygenic disorders, the number of genotypes could be equal to the number of patients.

## 2 Methods for Studying Combinations of Genetic Variants

### 2.1 Technical Methods

Genome-wide association studies and studies of selected genes can produce datasets that include billions of possible genetic variant combinations. Scanning and analyzing this huge amount of data can be impossible, even with relatively powerful computers. In addition to increased computer power, two technological developments have helped reduce the time needed to scan for combinations: massively parallel computing by graphics processing units (e.g., Nvidia GPU's) [1], [2], and cloud computing [3], [4].

Analyses of genetic variant combinations also require specialized software. For this purpose, algorithms and data mining tools have been developed based on methods such as regression analysis, Bayesian statistics, Boolean algebra, and array mathematics [5]. A recent review lists 27 publicly available applications for analyzing combinations of genetic data [6]. While some of these applications are complex, simple tools are also available. For example, the Excel function COUNTIFS can be employed to analyze combinations of only two genetic variants from a small variant pool [7].

Combinations occurring exclusively in patients can be obtained by analyzing combinations of 1, 2, 3, …, n SNP genotypes successively. Starting with the single SNP genotypes, those occurring exclusively in patients are selected and are not used for further combinations because they would all automatically be patient specific. The remaining SNP genotypes are now scanned for combinations of 2 SNP genotypes and those occurring exclusively in patients are selected and are not used for further combinations. The remaining combinations of 2 SNP genotypes are now scanned for combinations of 3 SNP genotypes and those occurring exclusively in patients are selected, and are not used for further combinations. In the end this procedure results in collections of single SNP genotypes, combinations of 2 SNP genotypes, combinations of 3 SNP genotypes, etc., all occurring exclusively in patients.

This procedure can be used with all types of genetic variants as well as with many clinical data.

### 2.2 Non-technical Methods

If a study of genetic variants includes too many combinations to allow analysis with the available technical tools, various methods can be applied to select smaller subgroups of combinations. For example, chi-square or similar tests can be used to analyze the distribution of each single genetic variant between patients and control subjects. Then the genetic variants with low *p* values can be paired with each other single variant to form two-variant combinations. This procedure can be repeated with the two-variant combinations to form three-variant combinations, and again with the three-variant combinations to generate four-variant combinations [8]. Similarly, biological criteria can be used to select single genetic variants of interest [9], which can then be used to form combinations with all of the variants.

Another way to drastically reduce the number of evaluated combinations is to analyze only combinations that are exclusively present in patients. This process would involve an initial scanning for combinations of genetic variants, followed by the selection of combinations occurring only in patients. Table 1 shows an example of combinations found exclusively in patients [10].

A total of 803 SNPs were analyzed for combinations of three SNP genotypes present in 607 bipolar patients and 1354 controls. Table 1 shows the distribution of the 57,911,211 combinations found exclusively in patients. Permutation tests revealed that all patient-specific combinations could be random findings, even the 1181 combinations that were common among nine or more patients. However, among these 1181 combinations, some clusters of combinations were significantly associated with bipolar disorder [10].

### 2.3 Statistics

For the analysis of polygenic disorders, chi-square, z-test or similar tests can be applied to determine whether the distribution of a genetic variant combination significantly differs between patients and control subjects. To assess whether combinations found exclusively in patients are significantly associated with the disorder, permutations tests can be performed, which are useful for analyzing many different genetic variant combinations selected from a dataset [11]. A permutation test can be applied to evaluate the assumption that genetic variant combinations present in many patients are more likely to be significantly associated with the disorder than combinations found in few patients. In a permutation test, the null hypothesis is that the observed data are exchangeable (permutable) with respect to groups—in this investigation, the patients and controls. This analysis involves the random re-distribution of indices for patients and controls, creating new groups of pseudo-patients and pseudo-controls of the same sizes as the original groups. This is repeated—for example, 1000 times—and the combinations found exclusively in pseudo-patients and common to many pseudo-patients are identified in each of the 1000 permutations. If the number of pseudo-patients harboring these combinations is the same or higher than in the original dataset in more than 50 of the 1000 permutations (*p* > 0.05), the null hypothesis is validated, suggesting that it may be a random occurrence that combinations were found exclusively in patients and were common to many patients.

In polygenic disorders showing pronounced genetic heterogeneity, there may be too few patients harboring the same combinations of genetic variants to confirm a statistically significant association between any single combination and the disorder. In such cases, statistical analyses can be performed using clusters or subgroups of many combinations. For example, a cluster can include selected combinations that contain a common SNP genotype. In another type of subgrouping, a chi-square test or z-test can be used to analyze the SNP genotype distribution between patients and controls, with the aim of selecting combinations that include an SNP genotype with a low *p* value. A third possible method is to select clusters in which each combination contains an SNP genotype related to a particular biological function or pathway [12]. If several clusters can be constructed from a sample of combinations, and each of these clusters is analyzed by a permutation test, the *p* values are corrected for multiple tests by the Benjamini-Hochberg correction [13].

Table 2 presents an example of a cluster, in which all of the combinations contain a common SNP genotype.

Table 2 shows an example of a cluster that is significantly associated with bipolar disorder. This cluster comprises 16 combinations of four SNP genotypes (from the 803 SNPs analyzed in Table 1). Among the 607 bipolar patients, 73 had at least one of these combinations in their genomes. These combinations were not found in the genomes of any of the 1355 control persons [8].

## 3 Combinations of Genetic Variants in Clinical Studies

Clinical studies of genetic variant combinations have primarily focused on potential associations between two-variant combinations and the disorder of interest. A review of several early studies did not find compelling statistical evidence validating the vast majority of reported interactions [6], and more recent studies support this conclusion [14], [15], [16]. A study including thousands of patients with breast cancer and control participants revealed no significant interactions among 2.5 billion possible two-SNP combinations [16]. Using an algorithm, APSampler [17], combinations of up to five genetic variants have been analyzed in studies of multiple sclerosis [18], [19]. And in networks of genetic variants, combinations of several genetic variants have been identified as associated with various disorders [20], [21], [22], [23].

None of the above-mentioned studies has separately studied combinations occurring exclusively in patients. In one investigation of bipolar disorder (summarized in Table 1), four clusters of combinations that were exclusively found in patients were significantly associated with bipolar disorder (*p* < 0.001). These four clusters contained 49, 46, 45, and 32 combinations, and combinations from these clusters were present in the genomes of 48, 37, 41, and 41 patients, respectively. One of these patient groups showed significantly more manic and depressive episodes than the other three groups [24]. Only 11 patients had combinations from two different clusters in their genome. A follow-up study investigated combinations of four SNP genotypes (summarized in Table 2), and found that a cluster containing 16 combinations was significantly associated with bipolar disorder [8]. A total of 73 patients showed some of these 16 combinations in their genomes, and 20 of these patients were also in one of the four above-described clusters with combinations of three SNP genotypes. Overall, 209 of the 607 patients with bipolar disorder had combinations from the identified clusters in their genome, whereas these combinations were not present in the genomes of any of the 1355 control participants.

Another study analyzed 16 SNPs in 370 patients with neuroblastoma and 803 control persons [25]. Scanning the material revealed 14,307 combinations of three SNP genotypes among these 16 SNPs. Of these combinations, 12,772 were common to both patients and controls, while 322 were found only in patients. A cluster containing 24 of these patient-specific combinations was significantly associated with neuroblastoma (*p* < 0.00001), and these combinations were present in the genomes of 32 patients with neuroblastoma. Among these 32 patients in the cluster, 20 (63%) were high-risk neuroblastoma cases, compared to a 43% proportion of high-risk cases among the 370 included neuroblastoma patients. This indicated enrichment of high-risk neuroblastoma cases within the cluster (*p* < 0.05).

In a study of oral cancer, 325 SNPs were analyzed in 373 patients and 535 control persons [7]. Scanning the material revealed 395,193 combinations of two SNP genotypes, including 328,238 combinations that were common to both patients and controls, and 46,469 present only in patients. Two clusters of patient-specific combinations were significantly associated with oral cancer (*p* < 0.001). Combinations from these clusters were present in the genomes of 205 of the 373 oral cancer patients, and not in the genomes of any of the 535 control persons. The two clusters contained 52 and 43 combinations, and were very different from each other, with no overlap between the represented SNP genotypes, indicating two completely different genetic subgroups of patients with oral cancers. One cluster contained combinations of SNP genotypes from a single biological pathway, and the patients in this cluster harbored relatively large numbers of these combinations in their genomes. The other cluster contained combinations from three different biological pathways, and patients in this cluster showed relatively few combinations in their genomes. These findings suggest that the accumulation of few genetic variants in several pathways can carry the same disease risk as the accumulation of many genetic variants in a single pathway.

## 4 Discussion

There are several methods of scanning a dataset of genetic variants for combinations of these variants. Small datasets can be directly scanned for combinations containing only a few variants. In larger datasets, it may be necessary to scan subsets of the variants to identify combinations. When a dataset is obtained from groups of patients and control persons, it can be helpful to separate the combinations occurring exclusively in patients from the combinations found in both controls and patients and those occurring exclusively in control persons.

Combinations occurring exclusively in patients may be significantly associated with the investigated disorder. However, in four studies of such combinations, no single combination was found to be significantly associated with the investigated disorder [7], [8], [10], [25]. Obviously, a combination that occurs only once in the study material will be present in either a patient or a control person, and such a combination will not be statistically significantly associated with a disorder. However, even combinations common among several patients and not present in controls are sometimes not found to be significantly associated with the disorder. This may be at least partly because the groups of patients having a common combination are too small to obtain statistical significance. To analyze larger groups of patients, it is sometimes possible to extract clusters of combinations that show some similarity, for example, where each combination in a cluster contains a common SNP genotype. Such clusters may show significant association with a disorder. Patients having one or more of the combinations from a cluster in their genome are considered to belong to that cluster. A prior study using this method found that up to 55% of patients had such combinations in their genomes, whereas none of the control subjects showed any of these combinations in their genomes [7].

Investigations of clusters of combinations occurring exclusively in patients have found that, although clusters are significantly associated with the disorder, individual combinations from these clusters do not show significant association with the disorder [7], [8], [10], [25]. These findings raise questions regarding the interpretation of a cluster of combinations that is significantly associated with a disorder. It is possible that a cluster of combinations that is significantly associated with a disorder could represent a general risk factor for the disorder, whereas the accumulation of combinations from the cluster in the genome of a patient may be regarded as a personal risk factor. In this respect, it would be interesting to assess whether the accumulation of many combinations in the genome results in higher risk or more severe disease, compared to the accumulation of fewer combinations from the clusters.

There are also unanswered questions regarding the generalizability of the findings from the few studies of combinations of genetic variants occurring exclusively in patients [7], [8], [10], [25]. Is it a coincidence that, in all four studies, the groups of patients harboring a common combination are too small for any single combination to achieve statistical significance? Or is this high degree of genetic heterogeneity typical for polygenic disorders? Answering this question will require more studies of combinations of genetic variants that occur exclusively in patients. Fortunately, it may be relatively easy to perform such studies as a supplement to new or ongoing studies, or by analyzing the genetic variants already reported in previous studies.

## Figures and Tables

**Table 1:** Scanning 803 SNPs for combinations of three SNP genotypes.

	Number of combinations of 3 SNP genotypes
Theoretical number with 803 SNPs, calculated as 803! / 3!(803–3)! × 3^3^	2,321,319,627
Found by scanning the material from 1354 control subjects and 607 bipolar patients	1,985,613,130
Common among both controls and patients	1,719,002,329
Found in 1354 control persons only	208,699,590
Found in 607 patients only	57,911,211
Found in single patients	45,285,770
Common among 2 patients	9,557,540
Common among 3 patients	2,277,107
Common among 4 patients	578,259
Common among 5 patients	156,343
Common among 6 patients	41,019
Common among 7 patients	10,990
Common among 8 patients	3002
Common among 9 patients	826
Common among 10 patients	261
Common among 11 patients	70
Common among 12 patients	22
Common among 13 patients	2
Common among ≥ 9 patients	1181

**Table 2:** A cluster of 16 combinations of four SNP genotypes.

	SNP1 genotype = YWHAH_rs1049583^c^ is found in all 16 combinations.		
	SNP2 genotype	SNP3 genotype	SNP4 genotype
1	TNC_rs1411456^b^	CNTN1_rs278913^b^	CNTNAP2_rs10272638^b^
2	TNC_rs1411456^b^	NFASC_rs2802853^b^	KCNQ3_rs10092250^a^
3	TNC_rs1411456^b^	CNTN1_rs11179168^b^	NFASC_rs9194^b^
4	TNC_rs1411456^b^	CNTN1_rs1056019^b^	CNTNAP2_rs10272638^b^
5	KCNQ2_rs6062929^a^	NRCAM_rs11974486^b^	MBP_rs8090438^a^
6	KCNQ2_rs6062929^a^	KCNN3_rs7547552^b^	ERBB4_rs707284^a^
7	KCNQ2_rs6062929^a^	MBP_rs12959623^b^	MAG_rs1034597^b^
8	ANK3_rs7906905^b^	SPTBN4_rs11672523^b^	KCNQ2_rs6011841^a^
9	CNTNAP2_rs10238991^b^	CNTN1_rs1056019^b^	KCNC1_rs1012105^a^
10	P2RX7_rs1718119^b^	IMPA2_rs3974759^b^	ANK3_rs10761454^a^
11	CNTN1_rs278913^b^	TNC_rs7035322^b^	CNTNAP2_rs10272638^b^
12	MBP_rs8090438^a^	CNTNAP2_rs2972112^a^	GSK3B_rs2037547^a^
13	KCNN3_rs7547552^b^	CNTN1_rs444927^b^	CNTNAP2_rs10464461^a^
14	CNTN1_rs278913^b^	CNTNAP2_rs17170126^b^	KCNN3_rs6426998^b^
15	SCN2B_SCN4B_rs645530^b^	ATP1A2_rs11585375^a^	NRCAM_rs11974486^b^
16	TNR_rs223982^b^	CNTNAP2_rs10277654^c^	NRG1_rs2466094^b^

a Wild-type homozygote.

b Heterozygote.

c Variant homozygote.
